# Gut Microbiome Characteristics in IgA Nephropathy: Qualitative and Quantitative Analysis from Observational Studies

**DOI:** 10.3389/fcimb.2022.904401

**Published:** 2022-05-17

**Authors:** Shisheng Han, Li Shang, Yan Lu, Yi Wang

**Affiliations:** ^1^ Department of Nephrology, Yueyang Hospital of Integrated Traditional Chinese and Western Medicine, Shanghai University of Traditional Chinese Medicine, Shanghai, China; ^2^ Institute of Science, Technology and Humanities, Shanghai University of Traditional Chinese Medicine, Shanghai, China

**Keywords:** gut microbiome, IgA nephropathy, systematic review, meta-analysis, observational study

## Abstract

**Background:**

Recent data indicate the importance of gut-kidney axis in the pathogenesis of Immunoglobulin A nephropathy (IgAN). Growing evidence suggests the alterations of diversity and composition of gut microbiome among patients with IgAN, however, the details are not yet fully understood.

**Methods:**

Eligible studies comparing the gut microbiome between patients with IgAN and non-IgAN individuals were systematically searched from PubMed, Embase, Web of Science, Cochrane Library, China National Knowledge Infrastructure, and *ClinicalTrials.gov*. The primary outcomes were alpha- and beta-diversity, and the differences in gut microbiota composition between patients with IgAN and non-IgAN persons. Qualitative analysis and meta-analysis were performed according to available data.

**Results:**

Eleven cross-sectional studies, including 409 patients with IgAN and 243 healthy controls, were enrolled. No significant differences in the diversity and enrichment of gut bacteria were found between IgAN and healthy individuals, whereas the beta-diversity consistently showed significant microbial dissimilarities among the two groups. *Firmicutes, Bacteroidetes, Actinobacteria, Proteobacteria, Fusobacteria*, and *Verrucomicrobia* were the dominant phyla, however, no significant differences were found between IgAN patients and healthy controls at the phylum level. The genera, *Streptococcus* and *Paraprevotella* showed a higher proportion in patients with IgAN compared to healthy individuals, whereas *Fusicatenibacter* showed a lower abundance according to meta-analysis. Qualitative analyses suggested that *Escherichia-Shigella* might be increased in IgAN patients; the genera, *Clostridium, Prevotella 9*,and *Roseburia*, members of *Ruminococcaceae* and *Lachnospiraceae* families, were likely to have decreased abundances in patients with IgAN compared to healthy individuals.

**Conclusion:**

Gut microbiota dysbiosis was demonstrated in IgAN, which might be involved in the pathogenesis of IgAN. Further studies are needed to confirm the findings of this study, due to the substantial heterogeneity.

**Systematic Review Registration:**

https://www.crd.york.ac.uk/prospero/, identifier PROSPERO (CRD42022304034).

## Introduction

Immunoglobulin A nephropathy (IgAN) is the most common immune-associated primary glomerulonephritis worldwide, characterized by the deposition of IgA, specifically, galactose-deficient IgA1 (Gd-IgA1), in the glomerular mesangium (Monteiro and Berthelot, 2021). Up to 40% of patients ultimately progress to end-stage kidney disease (Selvaskandan et al., 2022). Although the underlying pathogenesis has not been completely elucidated, the “multi-hit-hypothesis” is a widely accepted immunological interpretation for IgAN, that is, the overproduction of polymeric Gd-IgA1, anti-Gd-IgA1 and the formation of circulating immune complex containing Gd-IgA1, the mesangial deposition of Gd-IgA1 immunocomplex, and subsequent inflammation and fibrosis (Knoppova et al., 2021). Mesangial Gd-IgA1 deposits resemble mucosal IgA, mostly produced by mucosal B lymphocytes located in the Peyer’s patches, have been considered as an important pathogenetic factor of IgAN (Ohyama et al., 2021). Increasing evidence suggest a pivotal role of mucosal immunity in IgAN, which can be triggered by antigenic stimulation from the commensal microflora, more specifically, the gut microbiota dysbiosis and subsequent IgA production (Ichinohe et al., 2011). Intestinal mucosal hyperresponsiveness and abnormal production of Gd-IgA1 have been found in patients with IgAN, which was associated with specific fecal microbiota (Sallustio et al., 2021). The targeted-release of the glucocorticosteroid budesonide targeting excessive intestinal mucosal immune responses *via* releasing the drug to Peyer’s patches, showed a significant reduction of proteinuria compared to placebo in patients with IgAN (Fellström et al., 2017). Depleting of fecal microbiota by a broad-spectrum antibiotic prevented human IgA1 mesangial deposition, glomerular inflammation, and the development of proteinuria in a humanized mice model of IgAN (Lauriero et al., 2021). Patients with IgAN achieved partial remission after intensive fecal microbiota transplantation regularly for 6 months (Zhao et al., 2021). This evidence indicates the importance of gut-kidney axis in IgAN. Since the first human study by De Angelis et al. reporting the gut dysbiosis of IgAN (De Angelis et al., 2014), a growing number of studies have focused on the diversity and composition of gut microbiome in IgAN, however, the details are not yet fully understood. In addition, the gut microbiome is dynamic and differs in different populations, ages, sexes, seasonal variations, geographies, ethnicities, diets, and lifestyles (Gupta et al., 2017; Koliada et al., 2020; Koliada et al., 2021). Hence, this systematic review was conducted to comprehensively assess the diversity and abundance of the gut microbiome in patients with IgAN compared with non-IgAN individuals.

## Materials and Methods

### Design and Registration

This systematic review was registered prospectively at PROSPERO (CRD42022304034) and reported according to the Preferred Reporting Items for Systematic Reviews and Meta-Analyses (PRISMA) 2020 statement (**Supplementary Table S1**) (Page et al., 2021).

### Search Strategy

Eligible studies comparing the gut microbiome between patients with IgAN and non-IgAN individuals before March 1, 2022, were systematically searched from the following databases and registers: PubMed, Embase, Web of Science (WOS), Cochrane Library, China National Knowledge Infrastructure (CNKI), and *ClinicalTrials.gov*. A combination of MeSH with free text search was applied using the keywords gut microbiome, IgAN, and their associated subject words. The specific retrieval strategies are detailed in **Supplementary Table S2**.

### Eligible Criteria and Outcome Measures

Studies eligible for inclusion were original research that compared the diversity and composition of gut microbiome between biopsy-proven IgAN patients and healthy controls or non-IgAN persons. Exclusion criteria were as follows: (i) study included secondary IgAN or end-stage renal disease; (ii) unavailable data of gut microbiome.

The primary outcomes were as follows: (i) alpha- and beta-diversity; (ii) gut microbiome composition. Alpha diversity was evaluated to describe the community richness and diversity of gut microbiota. The Chao1 index, ACE index, and number of observed species/operational taxonomic units (OTUs) are estimated for microbial richness, whereas the Shannon and Simpson indices are calculated for microbial diversity. Beta diversity is a comparative analysis of microbial composition differences between patients with IgAN and controls. The secondary outcome of interest was the association of microbial signature and characteristics of IgAN.

### Study Selection and Data Extraction

The study selection and data extraction were independently performed by two reviewers (SS. H and Y. L) and disagreements were solved according to the decision of a third investigator (Y. W). The following data were extracted: first author, year of publication, location, design of study, baseline characteristics for all cohorts (sample size, age, sex, estimated glomerular filtration rate [eGFR] or serum creatinine level, urinary protein excretion), DNA extraction method, sequencing platform, bioinformatics pipelines, and outcomes.

### Quality Assessment

The Newcastle-Ottawa Scale (NOS) for the case-control study was adopted for quality assessment (Wells et al.). This scale consists of three dimensions with eight items, including selection, comparability, and exposure. The selection module contains four questions, including adequate definition of cases, representativeness of cases, selection of controls, and definition of controls. Comparability focuses on the controls of confounding factors. As the assessment of exposure, ascertainment of exposure, same method of ascertainment, and non-response rate should be answered. A maximum of nine scores can be awarded for a study, including one score for each item of the selection and exposure categories, and a maximum of two scores for comparability. A total score of ≥ 7 was considered as high quality (Suriyong et al., 2022).

### Data Synthesis and Analysis

For quantitative synthesis, the differences in bacterial diversity indices and relative abundances between patients with IgAN and non-IgAN individuals were estimated using standardized mean difference (SMD) and 95% confidence intervals (CIs). Heterogeneity was assessed utilizing Cochran *I* ^2^ test, which was considered significant when *P* < 0.10 or *I* ^2^ > 50% (Higgins et al., 2003). A fixed-effects model or a random-effects model meta-analysis was performed to calculate pooled SMD based on heterogeneity. Sensitivity analysis was conducted by omitting each study in turn.

For qualitative data analysis, the number of studies reporting statistically significant differences between IgAN group and control group for each prespecified outcome were recorded. We also carried out a funnel plot to compare the proportion of studies reporting significantly higher or lower relative abundances of specific intestinal bacteria, using the funnelR script and calculating a binomial Poisson distribution score2 with significant levels set at 50%, 80%, and 95% CIs (Woodall et al., 2022). Considering that several factors are associated with human gut microbiota composition, such as age, sex, seasonal variation, geography, ethnicity, diet, and lifestyle (Gupta et al., 2017; Koliada et al., 2020; Koliada et al., 2021), we further performed sensitivity analyses of matched confounding factors for the quantitative synthesis and qualitative analysis. Statistical analysis and graphical presentation were implemented by Stata (version 14.0), RStudio (Open source edition 2021.09.2), and GraphPad Prism (version 8.0).

## Results

### Characteristics of Included Studies

**Figure 1:**
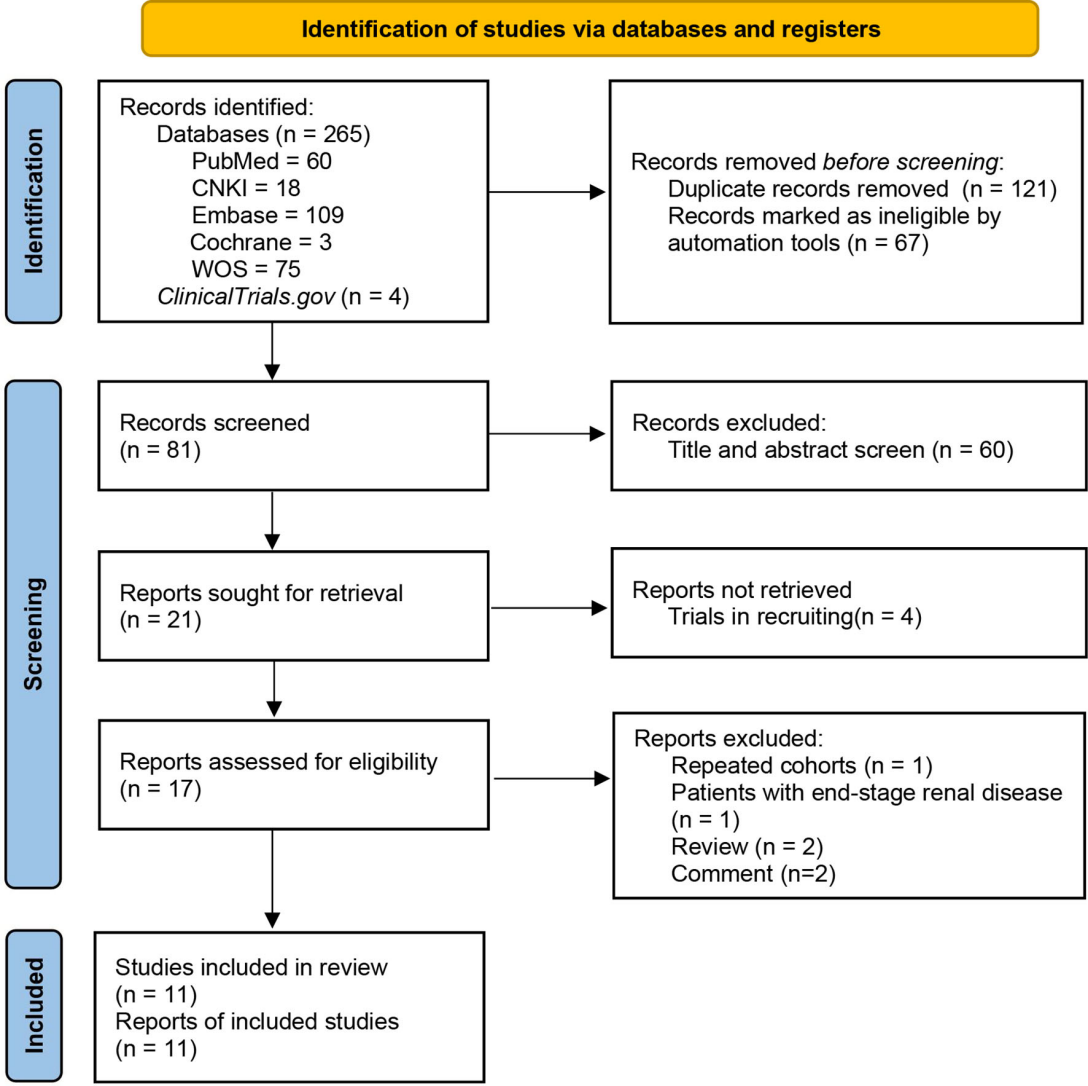
PRISMA flow diagram of study identification.

A total of 269 studies were retrieved from PubMed, Embase, WOS, CNKI, Cochrane Library, and *ClinicalTrials.gov*, 11 studies were finally included after eliminating duplications and screening in accordance with the pre-designed criteria (De Angelis et al., 2014; Dong et al., 2020; Hu et al., 2020; Zhong et al., 2020; Chai et al., 2021; He et al., 2021; Sugurmar et al., 2021; Tian et al., 2021; Weng et al., 2021; Wu et al., 2021; Ya et al., 2021). The detailed process of study identification is displayed in the PRISMA flow diagram (**Figure 1**).

The characteristics of included studies are described in **Table 1**, which were 11 cross-sectional studies published between 2014 and 2021, yielding 652 individual fecal samples for microbiome analyses. Nine studies were conducted in China (Dong et al., 2020; Hu et al., 2020; Zhong et al., 2020; Chai et al., 2021; He et al., 2021; Tian et al., 2021; Weng et al., 2021; Wu et al., 2021; Ya et al., 2021), one in Malaysia (Sugurmar et al., 2021), and one in Italy (De Angelis et al., 2014). All the gut microflora analyses were compared between patients with IgAN and healthy controls, who were adjusted with age and gender, additionally, body mass index (BMI) and dietary habits were also matched in seven studies (Dong et al., 2020; Hu et al., 2020; Chai et al., 2021; He et al., 2021; Sugurmar et al., 2021; Weng et al., 2021; Wu et al., 2021) and four studies (Hu et al., 2020; Zhong et al., 2020; Sugurmar et al., 2021; Wu et al., 2021). All the studies excluded the participants who were treated with antibiotics and/or probiotics within 1 to 3 months before stool collection. All the studies reported the collection and storage of fecal samples. Fresh stool samples were collected in containers and immediately stored at -80°C [five studies described “sterile” (De Angelis et al., 2014; Hu et al., 2020; Tian et al., 2021; Weng et al., 2021; Zhong et al., 2020)]. The amplified region of 16S rRNA gene (16S) was V3-V4 in nine studies (Dong et al., 2020; Hu et al., 2020; Zhong et al., 2020; Chai et al., 2021; He et al., 2021; Sugurmar et al., 2021; Tian et al., 2021; Wu et al., 2021; Weng et al., 2021) and V1-V3 in one study (De Angelis et al., 2014), one study did not specify the amplified region (Ya et al., 2021). Sequencing platforms from Illumina were used in nine studies (Dong et al., 2020; Hu et al., 2020; Zhong et al., 2020; He et al., 2021; Sugurmar et al., 2021; Tian et al., 2021; Weng et al., 2021; Wu et al., 2021; Ya et al., 2021). Ribosomal database project (RDP) was the most used bacterial and archaeal rRNA database for taxonomic assignments of sequence data (De Angelis et al., 2014; Dong et al., 2020; Hu et al., 2020; Zhong et al., 2020; He et al., 2021; Sugurmar et al., 2021; Wu et al., 2021).

**Table 1:** Studies characteristics and quality assessments of included studies.

Study	CountryDesign	Sample size (male %) Age (yrs), mean ± SD		eGFR (ml/min·1.73m^2^) or Creatinine^a^ (umol/L) Urinary protein (g/24h, g/g^b^)		Matched factors	Stool sample collection and storage	DNA extraction method (Region Amplified)	Sequencing platformDatabase used	Outcomes:Alpha-diversity index;Beta-diversity;Microbiome analysis	NOS score
		IgAN	Healthy controls	IgAN	Healthy controls						
De Angelis et al. (2014)	Italy Cross-sectional study	32 (66%) 43 ± 8	16 (60%) 43 ± 8	53 ± 28 0.59 ± 0.61	96 ± 7 0.05 ± 0.01	Age, gender	Fecal samples suspended in RNA later in sterile plastic box were stored at -80°C immediately	FastDNA Spin Kit for Soil 16S (V1-V3)	454 FLX Sequencer USEARCH, RDP	Observed *sp*., Chao1, Shannon; PCA; ANOVA	9
Zhong et al. (2020)	China Cross-sectional study	52 (46.2%) 35.0 ± 9.1	25 (48.0%) 31.5 ± 5.4	90.4 (68.0-116.1) 1.85 ± 2.10	116.9 (114.0-125.9) 0.06 ± 0.03	Age, gender, dietary habits and lifestyle	Fresh fecal samples were placed in sterile containers and immediately stored at - 80°C	E.Z.N.A.^®^ Soil DNA Kit 16S (V3-V4)	Illumina MiSeq UPARSE, RDP, SILVA	Observed *sp*., ACE, Chao1, Shannon, Simpson; PCoA; LEfSe, Wilcoxon rank-sum test, t-test	8
Dong et al. (2020)	China Cross-sectional study	44 (45.5%) 34.89 ± 10.74	30 (46.7%) 38.60 ± 12.80	73.30 ± 23.94 0.77 (0.38–1.37)	85.04 ± 18.24 NR	Age, gender, BMI	Fresh fecal samples were immediately stored at −80°C	E.Z.N.A^®^ Stool DNA kit 16S (V3-V4)	Illumina MiSeq UPARSE, RDP, SILVA	OTUs, ACE, Chao1, Shannon, Simpson; PCoA; LEfSe, Wilcoxon rank-sum test	9
Hu et al. (2020)	China Cross-sectional study	17 (82.3%) 44.76 ± 10.53	18 (77.7%) 49.72 ± 8.39	83.16+32.49 0.95 ± 0.81^b^	107.13+12.52 0.01 ± 0.001^b^	Age, gender, BMI, dietary habits	Fresh fecal samples were placed in sterile harvesters and frozen at −80°C in no more than 30 min.	AxyPrep DNA Gel Extraction Kit 16S (V3-V4)	Illumina HiSeq 2500 RDP	Observed *sp*., ACE, Chao1, Shannon; PCoA, UniFrac; Wilcoxon rank sum test	9
Chai 2021 (Chai et al., 2021)	China Cross-sectional study	29 (41.3%) 38.21 ± 11.80	29 (41.3%) 38.69 ± 9.90	101.95 (72.70, 123.44) 0.77 (0.36, 2.03)	103.89 (99.40,114.34) NR	Age, gender, BMI	Fresh fecal samples were collected in ice boxes, and transferred to -80°C within 30 min	PowerSoil^®^ DNA Isolation Kit 16S (V3-V4)	Ion S5^TM^ NR	Observed *sp*., Chao1, Shannon, Simpson; NMDS; LEFSe	9
Wu et al. (2021)	China Cross-sectional study	15 (46.6%) 38.64 ± 2.91	30 (66.6%) 44.1 ± 1.91	168.7 ± 61.26^a^ 1.94 ± 0.44	75.43 ± 3.24^a^ NR	Age, gender, BMI, dietary habits and lifestyles	Fecal samples were collected after overnight fasting, and stored at −80°C	PowerSoil DNA Isolation Kit 16S (V3-V4)	Illumina HiSeq PE250 USEARCH, RDP	OTUs, ACE, Chao1, Shannon, Simpson; PCoA; LEfSe, t-test	8
Sugurmar et al. (2021)	Malaysia Cross-sectional study	36 (36.0%) 45.5 ± 13.4	12 (33.0%) 46.5 ± 13.5	79.0 (62.1, 92.2) 0.44 (0.20-1.13)	86.5 (74.3, 93.8) 0.08 (0.08-0.08)	Age, gender, BMI, dietary habits	Stool samples were taken at home and brought to hospital within 6 h in cold storage and were stored at -80°C	GeneAll ExgeneTM Stool DNA Kit 16S (V3-V4)	Illumina NR QIIME RDP	OTUs, Shannon; NR; t-test and Mann-Whitney test	8
He et al. (2021)	China Cross-sectional study	Data set 1: 87 (52.87%) 38.78 ± 11.45	24 (50%) 35.79 ± 8.19	NR	NR	Age, gender, BMI	The fecal samples from each participant were collected in the hospital and immediately stored at -80°C	QIAamp Fast DNA stool minikit 16S (V3-V4)	Illumina HiSeq UPARSEH, USEARCH, RDP	Chao1; PCoA; Wilcoxon rank sum test	9
		Data set 2: 27 (80.0%) 44.68 ± 12.65	19 (42.1%) 31.32 ± 10.59	Without selection	Illumina MiSeqDx UPARSEH, USEARCH, RDP	7	
Weng et al. (2021)	China Cross-sectional study	40 (57.5%) 35.12 ± 6.48	10 (60.0%) 35.26 ± 7.09	65.76 ± 30.24 1.85 ± 1.69	103.73 ± 21.01 0.06 ± 0.03	Age, gender, BMI	Fresh fecal samples were collected in sterile containers and immediately stored at - 80°C	HiPure Bacterial DNA Kit 16S (V3-V4)	Illumina HiSeq4000 USEARCHGreenGene	Observed *sp*., ACE, Chao1, Shannon, Simpson; PCoA, UniFrac; LefSe, t-test, and Mann-Whitney test	9
Ya et al. (2021)	China Cross-sectional study	20 (50.0%) No difference in age	20 (50.0%) Data not presented	NR	NR	Age, gender	Fresh fecal samples were collected in cryogenic vials and immediately stored at - 80°C	FastDNA Spin Kit for Soil NR	Illumina Hiseq2500 NR	NR; NR; LefSe, t-test, and Wilcoxon rank sum test	8
Tian et al. (2021)	China Cross-sectional study	10 (30%) 44.50 ± 8.54	10 (30%) 42.70 ± 11.96	72.12 ± 14.18^a^ 1.51 ± 0.35	63.48 ± 10.90^a^ 0.07 ± 0.04	Age, gender	Fresh fecal samples were collected in sterile containers and immediately stored at -80°C	NR 16S (V3-V4)	Illumina Novaseq6000 UPARSE SILVA	Observed *sp*., ACE, Chao1, Shannon, Simpson; PCoA; LefSe, t-test, and Mann-Whitney test	8

SD, standard deviation; eGFR:estimated glomerular filtration rate; NR, not reported; BMI, body mass index; OTU, operational taxonomic units; Principal component analysis (PCA); PCoA, Principal coordinate analysis; NMDS, nonmetric multidimensional scaling; LEfSe, linear discriminant analysis effect size; UniFrac, analysis of similarity test; RDP, Ribosomal database project; QIIME, quantitative insights into microbial ecology; USEARCH,UPARSE, UPARSEH, SILVA: rRNA sequence databases. ^a^data of serum creatinine; ^b^data of urinary albumin-to-creatinine ratio.

All the studies consecutively included biopsy-proven IgAN, and at least matched age and sex between IgAN group and control group. Six studies were awarded NOS scores of nine (De Angelis et al., 2014; Dong et al., 2020; Hu et al., 2020; Chai et al., 2021; He et al., 2021; Weng et al., 2021). Five studies were given eight scores, because they did not report the details of recruitment for controls (Zhong et al., 2020; Sugurmar et al., 2021; Tian et al., 2021; Wu et al., 2021; Ya et al., 2021). Two independent cohorts were set in the study by He et al., which were the cohorts matched with age, sex, and BMI, and the other cohorts recruited without selection (He et al., 2021). NOS scores were assessed as 9 and 7, respectively.

### Primary Outcomes

#### Alpha- and Beta- Diversity

**Figure 2:**
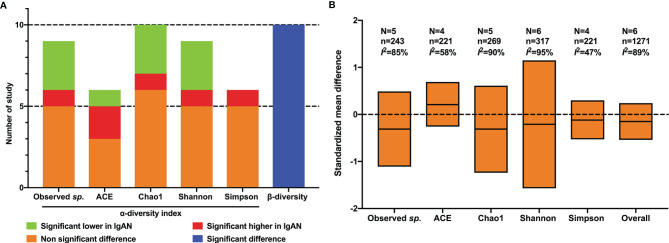
Qualitative analysis and meta-analysis of alpha- and beta- diversity of gut flora between IgAN and healthy control. **(A)** qualitative analysis; **(B)** meta-analysis; N, number of studies; n, number of participants.

At the individual study level, 10 studies reported the observed species or OTUs. Compared with healthy controls, only one study showed an increased observed species in patients with IgAN (Tian et al., 2021), three studies reported significant decreased OTUs (De Angelis et al., 2014; Dong et al., 2020; Hu et al., 2020), and unchanged OTUs were reported in five studies (Chai et al., 2021; Sugurmar et al., 2021; Weng et al., 2021; Wu et al., 2021; Suriyong et al., 2022). The ACE index was found to be significantly higher in IgAN than in control in two studies (Dong et al., 2020; Tian et al., 2021), lower in one study (Hu et al., 2020), and not significantly changed in three studies (Zhong et al., 2020; Wu et al., 2021; Weng et al., 2021). One study indicated a significant increase of the Chao1 index in patients with IgAN compared with healthy controls (Dong et al., 2020), three studies showed a significant decrease (De Angelis et al., 2014; Hu et al., 2020; Weng et al., 2021), and five studies with six cohorts reported no significant differences (Zhong et al., 2020; Chai et al., 2021; Wu et al., 2021; He et al., 2021; Tian et al., 2021). The Shannon index, reported in nine studies, was found to be significantly higher in IgAN patients compared to healthy controls in one study (Weng et al., 2021), lower in three studies (De Angelis et al., 2014; Chai et al., 2021; Wu et al., 2021), and not significantly changed in five studies (Dong et al., 2020; Hu et al., 2020; Zhong et al., 2020; Sugurmar et al., 2021; Tian et al., 2021). As for the Simpson index, five of six studies showed no significant differences between patients with IgAN and healthy controls (Chai et al., 2021; Dong et al., 2020; Tian et al., 2021; Weng et al., 2021; Zhong et al., 2020), a significant increase in the IgAN group was observed by Wu et al. (2021) (**Figure 2A**).

The results of meta-analysis showed that there was no significant difference in any of the alpha-diversity index between patients with IgAN and healthy controls: OTUs (SMD=-0.31, 95%CI -1.11, 0.49, *I* ^2 ^= 85%), ACE index (SMD=0.21, 95%CI -0.26, 0.69, *I* ^2 ^= 58%), Chao1 index (SMD=-0.31, 95%CI -1.24, 0.61, *I* ^2 ^= 90%), Shannon index (SMD=-0.21, 95%CI -1.57, 1.15, *I* ^2 ^= 95%), and Simpson index (SMD=-0.12, 95%CI -0.53, 0.30, *I* ^2 ^= 47%) (**Figure 2B**). Considering the substantial heterogeneity, we performed sensitivity analyses *via* by omitting each study in turn, the results were stable.

Different from alpha-diversity, significant microbial dissimilarities between IgAN and healthy controls were reported in 10 cohorts in terms of beta-diversity (**Figure 2A**), using principal component analysis (De Angelis et al., 2014), principal coordinate analysis (Dong et al., 2020; Hu et al., 2020; Zhong et al., 2020; He et al., 2021; Tian et al., 2021; Weng et al., 2021; Wu et al., 2021), and non-metric multidimensional scaling (Chai et al., 2021; Tian et al., 2021).

#### Microbial Composition at Phylum Level

**Figure 3:**
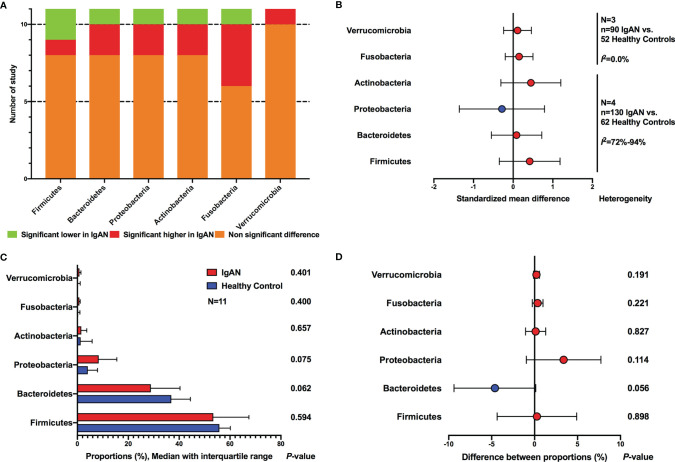
Comparison of gut microbial composition between IgAN and healthy control at the phylum level. **(A)** qualitative analysis; **(B)** meta-analysis; **(C)** Wilcoxon rank-sum test of average abundances at study level; **(D)** T-test of average abundances at study level.

All the included studies reported the intestinal microbial composition at the phylum level (**Figure 3A**). The relative abundances of six phyla accounted for more than 99% of the total community, including *Firmicutes, Bacteroidetes, Actinobacteria, Proteobacteria, Fusobacteria*, and *Verrucomicrobia*. For *Firmicutes*, only one study observed a significantly increased abundance in patients with IgAN (Weng et al., 2021), two studies reported significantly decreased proportions (Zhong et al., 2020; Ya et al., 2021), and eight studies showed no significant differences between IgAN and healthy individuals (**Figure 3A**). Regarding *Bacteroidetes*, no significant differences in the relative abundance were found between IgAN group and control group in eight studies, two studies observed significantly higher abundances in IgAN (Zhong et al., 2020; Weng et al., 2021), whereas one study reported a significantly lower proportion of abundance in patients with IgAN (Wu et al., 2021). *Actinobacteria* (Chai et al., 2021; Weng et al., 2021) and *Proteobacteria* (Dong et al., 2020; Wu et al., 2021) were reported to have significantly higher abundances in IgAN groups compared to the control groups in two studies, whereas a significantly lower proportion of relative abundance was found in IgAN patients in one study each (Zhong et al., 2020; Weng et al., 2021), no statistically significant differences were observed in the remaining studies. The relative abundances of *Fusobacteria* were found to be significantly higher among IgAN patients in four studies (Hu et al., 2020; Zhong et al., 2020; Sugurmar et al., 2021; Tian et al., 2021), whereas a lower proportion of abundance in IgAN group was reported in one study (Dong et al., 2020). For *Verrucomicrobia*, 10 studies did not find significant differences between sufferers of IgAN and healthy controls, and one study observed a higher abundance in IgAN group (Ya et al., 2021).

The meta-analyses on the basis of four studies also showed no differences between IgAN and healthy persons at phylum level (*Firmicutes*, SMD=0.42, 95%CI -0.35, 1.18; *Bacteroidetes*, SMD=0.09, 95%CI -0.55, 0.72; *Actinobacteria*, SMD=0.45, 95%CI -0.31, 1.20; *Proteobacteria*, SMD=-0.28, 95%CI -1.36, 0.79; *Fusobacteria*, SMD=0.15, 95%CI -0.20, 0.50; *Verrucomicrobia*, SMD=0.11, 95%CI -0.24, 0.46) (**Figure 3B**; **Supplementary Figure S1**). Sensitivity analyses indicated stable results, except that in *Actinobaheteria*. When one study was excluded (Tian et al., 2021), the synthetic estimate showed statistical differences (SMD=0.75, 95%CI 0.03, 1.47), however, the heterogeneity was still substantial (*I* ^2^ = 74%) (**Supplementary Figure S2**).

We also compared the reported average abundance of each phylum at the study level, using paired t-test and Wilcoxon rank-sum test. *Proteobacteria* was found to have a higher average proportion of abundance in patients with IgAN compared to healthy persons, and *Bacteroidetes* showed a lower abundance in IgAN, however, statistical differences were not reached (**Figures 3C, D**).

#### Microbial Composition at Genus Level

**Figure 4:**
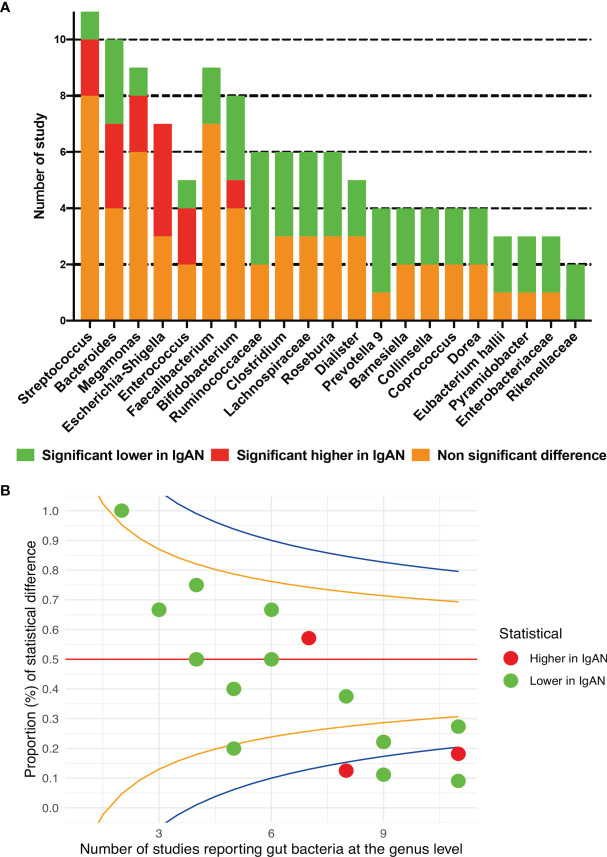
Gut microbial composition between IgAN and healthy control at the genus level. **(A)** qualitative description; **(B)** the funnel plot, specified score2 confidence limits are showed at 50% (red line), 80% (orange line) and 95% (blue line).

All the included studies reported the data of relative abundance between IgAN and healthy persons at the genus level. A total of 76 bacteria showed significant differences between the two groups. *Escherichia-Shigella* showed a higher relative abundance in patients with IgAN than in healthy persons in four studies (Dong et al., 2020; Hu et al., 2020; Ya et al., 2021; Zhong et al., 2020). Five genera, including *Clostridium, Prevotella 9*, *Roseburia*, members of *Ruminococcaceae* and *Lachnospiraceae* families, were found to be significantly lower in IgAN groups in at least three studies (**Figure 4A**; **Supplementary Table S3**). Opposite results were reported in five genera, including *Streptococcus, Bacteroides, Megamonas, Bifidobacterium*, and *Enterococcus*. The proportion of studies showed significantly changed abundances of each bacterium and the total number of reported studies did not exceed the upper 95%CI in any genus, according to the funnel plot (**Figure 4B**).

**Figure 5:**
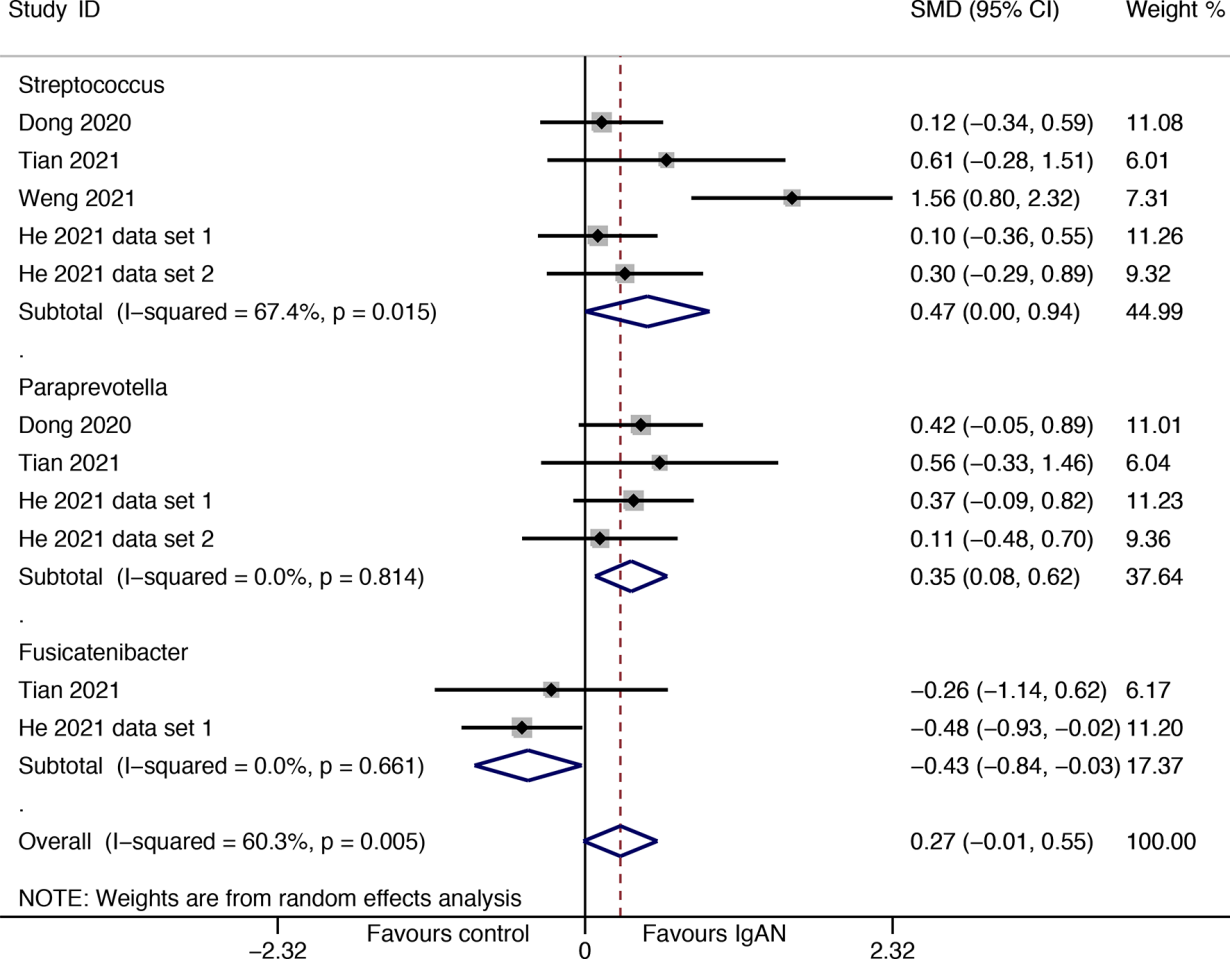
Forest plot for differences in gut flora between patients with IgAN and healthy controls.

Available data from four studies were used for meta-analysis, *Streptococcus* (SMD=0.47, 95%CI 0.001, 0.94) and *Paraprevotella* (SMD=0.35, 95%CI 0.08, 0.62) were found to have higher abundances in patients with IgAN than in healthy individuals; the genus *Fusicatenibacter* was found to have a lower proportion among IgAN sufferers than among healthy individuals (SMD=-0.43, 95%CI -0.84, -0.03) (**Figure 5**). Although the direction of estimates did not change, the statistical differences disappeared in sensitivity analyses for the three genera (**Supplementary Figure S2**).

### Secondary Outcome

Spearman correlation between fecal microbiota and clinical parameters of IgAN was reported in two studies (Dong et al., 2020; Hu et al., 2020). The genera, *Blautia, Veillonella, Anaerostipes*, and *Bifidobacterium* were found to have a positive association with eGFR, but *Escherichia-Shigella, Sneathia, Plesiomonas*, and *Defluviitaleaceae* were negatively correlated with eGFR. The enrichments of *Escherichia-Shigella, Sneathia, Parabacteroide, Defluviitaleaceae*, and *Anaerotruncus* were related to higher urinary protein excretion in IgAN, whereas *Rectale* was negatively correlated with urinary protein. Patients with hematuria <10/HP were found to have lower abundances of *Escherichia-Shigella* (Zhong et al., 2020). One study showed that *Prevotella-7* was negatively associated with Gd-IgA1 (Zhong et al., 2020).

### Sensitivity Analysis of Matched Confounding Factors

All the included studies have matched age and sex between patients with IgAN and healthy controls, and most studies (10/11) were conducted in Asia. Seven studies reported the timeline for recruiting subjects across summer and winter, and four studies did not describe the specific time of fecal sample collection. Therefore, we performed an analysis of intestinal flora in diet and lifestyle matched cohorts based on four studies (Hu et al., 2020; Zhong et al., 2020; Sugurmar et al., 2021; Wu et al., 2021). Non-significant differences in alpha-diversity and significant dissimilarities of gut bacteria between IgAN and healthy individuals were found (**Supplementary Figures S3A, B**). There were no significant differences in intestinal bacteria abundances between IgAN and healthy persons at the phylum level (**Supplementary Figures S3C–F**). *Escherichia-Shigella* showed a significantly higher abundance in patients with IgAN than in healthy controls in two studies (Hu et al., 2020; Zhong et al., 2020). Three genera, including *Prevotella 9*, members of *Ruminococcaceae* family, and *Coprococcus* were found to be significantly lower in IgAN in at least two studies (**Supplementary Figure S3G**). These results were consistent with the findings from the qualitative and quantitative analyses of all the included 11 studies.

## Discussion

The gut-kidney axis comes central to the pathogenesis in IgAN, and gut dysbiosis has been proven closely associated with IgAN (Coppo, 2018). This is the first systematic review comparing the differences in gut microbiome between patients with IgAN and healthy individuals involving 11 studies and 652 participants. Although we did not find significant differences in the diversity and enrichment of intestinal bacteria according to the alpha-diversity indexes of OTUs, ACE, Chao1, Shannon, and Simpon, the beta-diversity consistently showed significant microbial dissimilarities between IgAN and healthy persons, indicating gut dysbiosis of IgAN. More specifically, at the phylum level, we found an increase of *Proteobacteria*, but a decrease of *Bacteroidetes* among patients with IgAN, which is consistent with the subgingival microbiome of IgAN sufferers (Cao et al., 2018), although the difference was not statistically significant. At the genus level, *Streptococcus* and *paraprevotella* showed a higher proportion in cases of IgAN compared to healthy individuals, whereas, *Fusicatenibacter* showed a lower proportion according to meta-analysis. Qualitative analyses suggested that *Escherichia-Shigella* might be increased in IgAN patients because four studies reported higher relative abundances and three studies showed no significant differences when compared with healthy controls. The genera, *Clostridium, Prevotella 9*, *Roseburia*, members of *Ruminococcaceae* and *Lachnospiraceae* families, were found to be decreased in patients with IgAN in at least three studies, but no reports of increased abundances compared to healthy individuals.

The enrichment of *Proteobacteria* is considered a potential microbial diagnostic signature of dysbiosis and increases the risk of host diseases (Shin et al., 2015). *Proteobacteria* was also found to have a higher abundance of circulating microbiome profile in patients with chronic kidney disease (CKD) than healthy controls and correlated inversely with eGFR (Shah et al., 2019). Additionally, the abundances of *Proteobacteria* were depleted in two cases with IgAN, which showed alleviation of proteinuria after fecal microbiota transplantation (Zhao et al., 2021). The abundances of *Streptococci, Paraprevotella*, and *Escherichia-Shigella* probably increased in gut microbiota of patients with IgAN according to our meta-analysis and qualitative analysis. *Streptococcal* antigens binding with IgA were found to be deposited in renal tissue of patients with IgAN (Schmitt et al., 2010); additionally, IgAN is often induced or aggravated after suffering upper respiratory tract infection or gastrointestinal tract infections. This evidence indicated the potentially harmful role of *Streptococcus* in the pathological mechanism of IgAN. *Escherichia-Shigella* is a gram-negative, oxidase-negative, rod-shaped bacterium from the *Proteobacteria* phylum, which can result in intestinal infection under conditions (Wu et al., 2020; van den Beld et al., 2022). An increase of *Escherichia-Shigella* may cause local infection and activate gut immune responses, leading to the excessive synthesis of IgA (Tao et al., 2019). Moreover, patients with IgAN enriched with *Escherichia-Shigella* in the gut had higher urinary albumin excretion rate, worse hematuria, and lower eGFR (Hu et al., 2020; Zhong et al., 2020). The increase of *Escherichia-Shigella* also exacerbated gut leakiness by reducing butyrate biosynthesis and increasing oxidative stress to penetrate the intestinal epithelial barrier (Croxen et al., 2013). *Paraprevotella* was also found to be enriched in the fecal samples of patients with CKD, and to be superior in discriminating CKD from the healthy individuals (Li et al., 2019). Many intestinal bacteria have been shown to be associated with the production and metabolism of various short-chain fatty acids (SCFA). SCFAs have been documented as having important roles in maintaining health, such as acting as a nutrient source of the gut epithelium, protecting the intestinal mucosal barrier, and inhibiting inflammation (Zhang et al., 2015). SCFAs, especially acetate and butyrate, were found to inhibit the proliferation of glomerular mesangial cells and oxidative stress induced by lipopolysaccharides and high glucose *in vitro* (Huang et al., 2017). Patients with IgAN and CKD were found to have decreased levels of SCFAs (Felizardo et al., 2019). Some genera with lower abundances in cases of IgAN compared with healthy controls, according to our results, including *Clostridium, Prevotella 9*, *Roseburia*, members of *Ruminococcaceae* and *Lachnospiraceae* families, were confirmed as important bacteria involving the production and metabolism of SCFAs (Baxter et al., 2019; Fei et al., 2020; Sun et al., 2022; Wu et al., 2020; Xie et al., 2021). *Barnesiella* is one of the most abundant genera that has anti-inflammatory protective effects, which were also decreased in patients with IgAN, according to our results (Weiss et al., 2014). A chimeric fusion of Fc segment of human IgG1 and AK183, which is an IgA protease from the genus *Clostridium*, was found to promote renal clearance of IgA and obliteration of blood IgA and remove C3 deposits in the glomerulus (Xie et al., 2022). Therefore, the findings of this study demonstrated the gut dysbiosis of IgAN, characterized by an increase of pathogenic bacteria and a decrease of beneficial bacteria, especially the SCFA-associated species.

The strength of this systematic review is that we conducted a comprehensive search to ensure all relevant studies reporting the gut microbiome between IgAN and non-IgAN individuals. All the included studies adopted high throughput 16S sequencing to analyze the composition of intestinal flora. Additionally, patients who took medications that might result in modifications of gut microbiome before fecal specimen collection were excluded from all the selected studies. All the included studies had a NOS score of ≥ 8, suggesting high methodological quality.

Several limitations should be considered. First, the sample sizes of included studies are relatively small, which might lead to more uncertainty and less precision in the findings. Second, meta-analyses can only be performed among five studies, because some studies did not report sufficient data on the diversity and relative abundance of the gut microbiome for quantitative synthesis, therefore, the results of this review might not be able to fully reflect the current evidence. Third, we did not perform a subgroup analysis stratified by ethnicity and pathological severity, due to the limited data, although these factors may influence the composition of intestinal flora.

## Conclusions

In conclusion, this study presents a comprehensive analysis of the intestinal microbiota in patients with IgAN, and showed significant differences in gut bacterial composition between IgAN and healthy individuals. Due to the potential limitation and substantial heterogeneity, high-quality studies with large sample sizes are needed to confirm the detailed gut dysbiosis of IgAN.

## Author Contributions

LS and YW designed this study; SH and YL conducted the literature search, data extraction, quality assessment; SH completed data analyses and manuscript writing. All authors approved the final manuscript.

## Funding

This study was supported by the Science and Technology Commission of Shanghai Municipality, China (grant number 20Y21902100) and three-year projects for TCM development in Shanghai [grant number ZY (2018-2020)-FWTX-4027].

## Conflict of Interest

The authors declare that the research was conducted in the absence of any commercial or financial relationships that could be construed as a potential conflict of interest.

## Publisher’s Note

All claims expressed in this article are solely those of the authors and do not necessarily represent those of their affiliated organizations, or those of the publisher, the editors and the reviewers. Any product that may be evaluated in this article, or claim that may be made by its manufacturer, is not guaranteed or endorsed by the publisher.
